# A Digital Pornography Education Prototype Co-Designed With Young People: Formative Evaluation

**DOI:** 10.2196/65859

**Published:** 2025-03-04

**Authors:** Jake Turvey, Michelle Raggatt, Cassandra J C Wright, Angela C Davis, Meredith J Temple-Smith, Megan S C Lim

**Affiliations:** 1 Young People's Health Research, Burnet Institute, 85 Commercial Rd, Melbourne, 3004, Australia; 2 School of Public Health and Preventive Medicine, Monash University, Melbourne, Australia; 3 Alcohol and other Drugs team, Menzies School of Health Research, Casuarina, Australia; 4 Department of General Practice and Primary Care, University of Melbourne, Melbourne, Australia; 5 Melbourne School of Population and Global Health, University of Melbourne, Parkville, Australia

**Keywords:** pornography, education, website, prototyping, evaluation, sexual health, sexual wellbeing, pornography literacy, young people, youth, adolescents, The Gist, sexual education, Australia, efficacy, digital health, co-design

## Abstract

**Background:**

Interventions to help young people make sense of sex and relationships in the context of widely available pornography are becoming increasingly supported in school settings. However, young people who experience disruptions to their education often have less access to such programs. Digital platforms may offer a more accessible method to deliver tailored sexual health and pornography literacy to young people who are disengaged from mainstream schooling, or who experience other types of structural disadvantage.

**Objective:**

This study aimed to describe the formative evaluation of “The Gist” a co-designed online sexual health education and pornography literacy prototype designed to meet the sexual health information needs of structurally marginalized young people in Australia.

**Methods:**

We conducted iterative workshops with 33 young people aged between 15 and 24 years recruited from an alternative education school in Melbourne, Australia. Through interactive activities, participants evaluated the overall prototype design, including its usability, desirability, inclusiveness, and potential for impact.

**Results:**

Participants reported The Gist to be easy to use (17/20, 85%) and safe (19/23, 83%), with “hot” branding (25/30, 83%). However, perceived content relevance was dependent on the participants’ existing level of sexual health knowledge and experience, with only 31% (7/23) agreeing that “The Gist feels like it was made for me.” The interactive learning activities such as the debunked (myth-busting) and quiz features were among the most used and well-liked on The Gist platform. Low unprompted engagement with the prototype outside of facilitated workshop settings also confirmed previous researcher postulations that The Gist as a standalone digital platform is unlikely to meet the needs of this population group. Further design refinements are needed to improve user experience, including more interactive activities and visual information in place of heavily text-based features.

**Conclusions:**

This study provides important insights into the design and sexual health information needs of structurally marginalized young people. Further research is needed to assess the overall efficacy of The Gist prototype, as well as its ability to positively influence young people’s sexual attitudes, beliefs, and behaviors. Future iterations should consider hybrid or face-to-face delivery models to better capture student engagement.

## Introduction

### Pornography and Pornography Literacy

Pornography is easily accessible to, and commonly viewed by young people. In a 2017 cross-sectional survey of 941 Australians aged 15‐29 years, 84% of males and 23% of females viewed pornography at least weekly [1]. Some young people report that pornography is an important component of their sexual development [2], helping to define understandings of sexual pleasure, how to have sex, and diverse sexual experiences and sexualities [3]. However, pornographic content also commonly includes behavior which portrays problematic themes, including the promotion of heteronormative and misogynistic gender norms, violence-supportive sexual objectification of women, lack of consent, and the poor representation of diverse people and body types [4]. Research has further shown that pornography can shape young people’s sexual development in line with these problematic themes, and negatively influence their developing attitudes, beliefs, and sexual health behaviors [2,5,6].

Pornography literacy education is a technique which aims to reduce the adverse impacts of pornography use. Originating from media literacy theory, pornography literacy programs seek to increase young people’s knowledge and understanding of healthy relationships and sexualities, whilst developing their ability to critique, evaluate, and challenge the various themes commonly present within pornographic content [7]. However, there remains a scarcity of formally evaluated pornography literacy interventions [8-10]. In a 2018 pilot study conducted in the United States, it was observed that students demonstrated improvements in various pornography-related knowledge items after participating in a college-based pornography literacy class [11]. Similarly, a longitudinal study of Dutch teenagers found that the relationship between pornography use and sexist attitudes became weaker the more a participant engaged in school-based pornography literacy classes [12]. These findings therefore suggest that there is potential for pornography literacy programs to shape the way in which young people perceive common underlying themes and messages within online pornographic content [12]. While pornography literacy programs show promise in mitigating the adverse impacts of pornography on young people, determining how best to integrate and deliver these programs within existing educational frameworks is crucial for their success.

### Relationships and Sexuality Education

School-based sex education initiatives have traditionally been the preferred approach to improving sexual health outcomes amongst young people and mitigating risks of sexual health related harms such as sexually transmitted infections (STIs) and unplanned pregnancy [13]. In Australia, the delivery of relationships and sexuality education (RSE) has evolved in recent decades to include a broader range of contemporary sexual health topics and themes [14]. However, creating programs that adequately cater to the complex sexual health needs of diverse young people has posed an ongoing challenge for Australian educators and policymakers alike [15]. Furthermore, the actual education received by students is highly varied nationwide, as the development and implementation of RSE curricula remains the responsibility of each individual state or territory, while content delivery often differs between individual schools [14]. In a 2018 national survey of 6327 Australian high school students, more than 60% of respondents described their RSE experience as either irrelevant or marginally relevant to their sexual health needs, with many advocating for a greater focus on sexual communication, relationships, and pleasure [16]. The Australian government introduced mandatory consent education from 2023, covering areas of sexual consent, gender stereotypes, and power and relationship dynamics. However, there have been growing calls for the further addition other contemporary topics (such as pornography literacy) that take a more sex positive approach, and are more inclusive of alternative sexualities, gender identities, and other intersectionalities [17-19].

Furthermore, school-based education may not meet the needs of all young people. Young people have many different intersecting experiences that mean they have more or less opportunities for healthy sexual education and development. Young people who are disengaged from, or experience disruptions to their mainstream education or for example, have less access to school-based sex education programs [20]. In addition, school-based sex education is often not tailored to meet the specific needs of young people who are lesbian, gay, bisexual, transgender, queer, questioning, intersex, asexual (LGBTQIA+), young people from diverse cultural backgrounds, or young people with experiences of poverty [21-23]. International research also indicates that young people with experiences of trauma, family conflict, and other adverse childhood experiences have poorer access to sexual health information compared with other mainstream cohorts, and have an increased likelihood of engaging in risky sexual health behaviors [24]. Interventions to support young people’s sexual development in the context of pornography therefore, need to account for this substantial variation in sexual health development among young people, and must consider the specific needs of individuals who have been excluded from current relationships and sexuality education.

Digital platforms have shown increasing promise as an acceptable way to reach and deliver tailored sexual health information to young people [25]. Recent systematic reviews have highlighted their effectiveness at shaping young people’s knowledge of healthy sexual development, and reducing risky sexual health behaviors [25,26]. However, many of these interventions remain highly focused on singular or complementary behavioural outcomes in adolescents, such as STI prevention and contraception use [26]. As such, there is a distinct need for an online sexual health platform that is not only informed by, and tailored to the specific needs of structurally marginalized young people, but also provides them with a more comprehensive education on a wide range of sexual health topics, including pornography literacy.

### Aims

The Gist is an online relationships- and sexuality-education platform, incorporating elements of pornography literacy, that was co-designed by young people, sexual health researchers, educators, and web developers [4]. Here, we describe a formative evaluation of The Gist prototype, an evaluation designed to pretest a program with the target group before implementation [27]. This evaluation aims to assess The Gist’s usability, desirability, inclusiveness, and potential for impact, as well as identifying areas for future improvement.

## Methods

### The Intervention

Development of The Gist used a human-centred and co-design methodology [4]. Several months before this study, young people who identified as LGBTQIA+, were culturally and linguistically diverse, had experiences of family conflict or educational disengagement participated in a series of iterative workshops to provide in-depth insights and design ideas for a sexual health education resource. Based on their insights and design recommendations, a minimally functioning digital prototype was developed. This is a working website that demonstrated most of the planned features of The Gist with some of the planned content. This study aimed to test and further refine the prototype design. To give context to this evaluation, the key design considerations and features of The Gist are briefly described below. More in-depth information about the methodology and findings from the design and development phase is available in a separate publication [4].

To give context to this evaluation, the key design considerations and features of The Gist are briefly described below. More in-depth information about the methodology and findings from the design and development phase is available in a separate publication [4].

The Gist prototype was designed as an online website application, with mobile-friendly capacity. Topics covered included pornography, body diversity, sex, consent, pleasure, healthy relationships, and sexual preferences. Language and imagery remained gender- and sexuality-neutral. Critical pornography literacy was built into this content by contrasting the healthy and ethical information about sex, relationships, and bodies with problematic messages often espoused in pornographic materials. The visual design of the digital prototype had an alternative, “retro 90s” and comic-book style as preferred by participants from the earlier co-design stage.

The website prototype presented short facts about various sexual health topics to engage, surprise and challenge the user. Users were then linked to more in-depth information about the topic, and encouraged to engage with interactive activities that further challenged their knowledge and built critical thinking skills around the respective topic. Key platform features included:

1. Get Facts: a series of rolling screens presented a short fact with an image, designed to reproduce the “stories” function used on social media apps such as Instagram and Snapchat.
2. Debunked*:* presented users with a short statement on a series of sexual health topics and prompted users to decide whether the statement was true or false. This feature was designed to challenge commonly held misconceptions and beliefs about certain sexual health topics.
3. Quizzes: brief interactive quizzes presented through multiple-choice questions designed to test the user’s knowledge of key sexual health themes.
4. Sexual Health Articles: provided in-depth written information on various sexual health topics broken down into digestible subsections.

### Evaluation Questions

This paper reports on the testing and evaluation phase of the human-centered design process, which is part of an iterative cycle of prototyping, testing, and refining. The insights from this evaluation phase will be used to further refine and develop The Gist prototype for improved application. The key evaluation questions were: (1) is The Gist usable and accessible for young people?, (2) is The Gist desirable and relevant to young people?, (3) is The Gist inclusive and safe for young people?, and (4) do young people believe that The Gist has potential to positively impact other young people’s knowledge, attitudes, and confidence around respectful and healthy relationships, sexual health and wellbeing, and pornography literacy?

### Recruitment and Setting

Evaluation participants were recruited from across 3 campuses of an alternative education school in Melbourne, Australia. Students at this school have experiences of fragmented school attendance or disengagement from mainstream school settings for a range of reasons including family conflict or breakdown, bullying, being gender and sexually diverse, living with a disability, or being culturally and linguistically diverse. Researchers first spoke to school staff, providing them with an overview of the study and demonstration of The Gist prototype so that teachers were informed of the content that would be shown to students, and could voice any concerns about certain topics that some students may find distressing. After feedback and strong support from staff was received, the research team then spoke to students, and provided a brief overview of the study, workshop activity and content themes, before distributing plain language participant information and consent forms to those interested.

### Participants

A total of 33 young people participated in at least 1 of 7 workshops held across a 3-week period. Of the 33 participants; 15 identified as female, 13 identified as male and 1 identified as nonbinary (4 participants elected not to provide demographic information). Participants’ ages ranged from 15‐24 years with a median age of 19 years. A total of 18 participants identified as heterosexual, 11 identified as LGBTQIA+, and 8 spoke a language other than English or in addition to English at home.

### Workshop Methodology

In total, 3 groups of students across 2 campuses attended a series of 2 workshops each. Only 1 workshop was carried out at the third campus as these students had previously participated in the initial co-design research process for The Gist earlier in the year and were therefore already familiar with The Gist prototype.

All workshops were facilitated by 3 members of the research team, including 1 digital designer and 2 public health researchers. Two of these facilitators had been involved in the development of the prototype, all identified as young women (age<35 years old). School staff were present in 1 workshop (teacher supervision was required due to an incident earlier in the day unrelated to the research project). The duration of the workshops ranged from 70‐135 minutes. An overview of the workshop aims is available in . Workshop activities were specifically designed to be engaging, interactive, and trauma-informed, due to the unique learning and classroom needs of the participants. A total of 10 different activities were used across the various workshops, with a descriptive overview of each workshop activity available in .

### Data Collection

A range of qualitative and quantitative data was collected from across the 7 workshops.

#### Qualitative Data

Qualitative data included audio recordings of workshop activities and classroom discussions and physical material produced by students during workshop activities (ie, images of whiteboard brainstorming and completed worksheet activities). One researcher recorded written observations on classroom activities, levels of student engagement, and overheard classroom discussions. They used a semistructured note-taking guide developed by the lead researcher, to supplement audio recording with observations of nonverbal interactions. In addition, while students explored the prototype, 1 participant in each group (n=3) volunteered to be individually observed to collect more detailed information on website navigation and interactions. The facilitator recorded their actions in detail including which buttons were pressed and which features were accessed.

One researcher recorded written observations on classroom activities, levels of student engagement, and overheard classroom discussions. They used a semi-structured note-taking guide developed by the lead researcher, to supplement audio recording with observations of non-verbal interactions.

In addition, while students explored the prototype, one participant in each group (n=3) volunteered to be individually observed to collect more detailed information on website navigation and interactions. The facilitator recorded their actions in detail including which buttons were pressed and which features were accessed.

#### Quantitative Data

Quantitative data included surveys conducted during workshops explored students’ receptiveness to The Gist prototype, including measures on usability and accessibility, desirability and relevance, safety and inclusivity, and potential to impact (). A five-point Likert scale was used to assess survey responses, with response options ranging from 1= “Nah Shit,” 2=“Pfft,” 3=“Meeh,” 4=“It’s OK,” 5=“Love it!!!.” Students used stickers to indicate their responses on a large poster displaying the questions and website analytical data including unique page visits.

Website analytical data including unique page visits.

### Data Analysis

This research was embedded in the constructivist paradigm. Following initial data cleaning and familiarisation, author [MR] developed a thematic framework guided by the evaluation questions, as well as emerging themes from initial data familiarisation processes. This framework was then applied to the data by authors [MTS and MR] by coding the observer notes, audio recordings, and images directly into NVivo 12 software (Lumivero). Researchers then summarized emerging themes into charts and tables to visualize the data as a whole, before mapping and synthesising differences across different participant groups and workshops. Throughout each phase, the thematic framework was refined as new themes or subthemes emerged.

Simultaneously, the quantitative data sourced from workshop activities and the workshop surveys were analysed by author [MR] using descriptive statistics. Then, a narrative integration approach was used to weave both qualitative and quantitative findings together on a theme-by-theme basis by authors [JT, MR, CW, and ML]. To achieve this, the quantitative findings were mapped to the thematic framework, and used to support or expand upon the emerging study themes, providing a stronger and more comprehensive understanding of the themes. Findings and key themes are arranged according to the evaluation questions. All supporting quotes are presented anonymously.

### Ethical Considerations

Ethics approval for the research was granted by the Alfred Health Human Research Ethics Committee (373/18). All participants provided written informed consent. For participants under the aged of 18 years or younger, written or verbal parental consent confirmed via school staff was sought before workshop commencement. All data have been deidentified. Participants received a AUD20 (USD 13.90) voucher per workshop, plus a AUD10 (USD6.95) voucher if attending both workshops. Participants who were already familiar with the prototype and were only invited to a single workshop received a AUD25 (USD17.40) voucher.

## Results

### Usable and Accessible

#### Use and Usability

**Figure 1.**
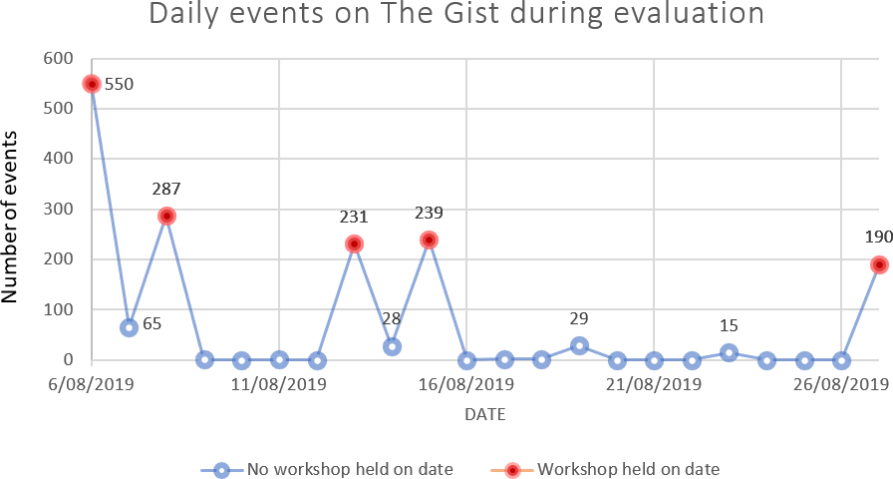
Daily unique page visits on The Gist website during the evaluation period.

In general, participants were able to access and engage with The Gist website effectively, with 85% of workshop participants (17/20) responding with either “Love it!!!” or “It’s OK” when asked if “The Gist is easy to use.” However, website analytical data suggests that actual engagement with The Gist outside of the facilitated workshops was extremely low. Of the 1641 unique page visits recorded across the evaluation period, 1497 (91%) were recorded during workshop sessions (Figure 1), suggesting few participants independently engaged with the website.

Researcher observations also noted that students primarily engaged with The Gist in small groups during workshop sessions, with many participants preferring to explore the website features alongside their peers, rather than as an individual endeavor. Students seemed to value the shared discussions which were stimulated by The Gist content and engaged each other in a collective learning process.

#### Non-Intuitive Design Features

Participants also reported design issues that meant several of The Gist features were not as intuitive or easy to use as participants would have liked. The Get Facts feature for example, was not clearly identifiable as content, with several participants initially attempting to use it as a navigation function. The ability to filter content within the search function, was not obvious to many participants who would either scroll past this option or requested clarification from facilitators.

It’s all organised but I wouldn’t really know how to go around using it such as what the app offers. But after a while at using it I would get used to it

#### Further Information and Learning

Participants were receptive to the different interactive activities available. Over the evaluation period, the most popular and well used features were “Debunked” (302 events), “Quizzes” (127 events), and “Get Facts” (100 events). The home page received 240 visits and “Articles” were viewed 84 times.

I first checked “Get Facts” tab section. Informative, liked the flicking facts.

When you click something and then you learn something, it’s not complicated. The Get Facts [feature] works

Articles were generally the least used learning feature, owing largely to their length and heavy use of text over interactive content and images, despite our attempts to prioritise brevity. Several participants commented that they did not enjoy reading, and quickly abandoned the feature or skipped over the articles altogether, preferring to access information through more interactive means. This is despite feedback from participants suggesting that the content did not go far enough with the information presented, with participants wanting the opportunity to learn more about specific topics that interested them. However, amongst the participants who reviewed the article feature, the majority (17/21, 81%) enjoyed (ie, “it’s OK” or “Love it!!!”) the format of the articles which used subheadings to break down relevant information, while 95% (20/21) thought the articles were easy to read. While a dot point summary of each article was provided, this was placed at the end of the article, with facilitators once again observing many participants either abandoning the article before reaching the summary, or simply scrolling past.

For me, because I don’t like reading, I would say the articles weren’t very important to me. But that’s because I hate reading.

I really love the [article] layout and the expandable boxes – the word I’d use is nifty.

The articles were easy to read, [and] I did learn some cool things about consent.

### Desirable and Relevant

#### Content Relevance

Overall, participants held generally positive opinions of The Gist and its content, with 68% (15/22) of workshop participants agreeing with the statement, “The Gist is definitely something I would use.” More than 73% (16/22) of participants also agreed that they “learnt some cool stuff from the Gist.” Many participants reflected that The Gist was both helpful and informative, with the features of the website serving as effective learning strategies to deliver new and engaging sexual health information.

Yes [relevant] because some of these things happen in my life

Participants particularly enjoyed how the content was specifically catered toward young people, and sought to openly discuss topics that were frequently absent from within other mainstream sexual health education programs.

I really liked how The Gist is better than any sex education you get in school

It’s more appropriate than other sites because it’s made for young people that don’t understand sex ed at school or just want to learn.

…get info about things that are not typically spoken about

I like that it was open and uncensored

However, while most participants felt that The Gist was relevant to young people in general, several participants reflected that the prototype was not overly relevant to them personally. Two opposing explanations were offered for this, with some participants suggesting that The Gist’s content was more appropriate for a younger audience, with quizzes being “too easy,” and that they already knew much of the information presented. In contrast, several other participants reflected that much of the content was too advanced for them, especially as they had little previous knowledge or sexual experience. They explained that there was not enough information for “beginners” who were not comfortable learning about sex-based topics. Similar apprehensions were expressed by a small number of students who identified as asexual, or those who had never engaged in watching pornographic material.

I probably won’t use it that much because the info is stuff I mostly know.

More information for younger/less experienced.

Survey results supported this mixed response. When asked if “The Gist feels like it was made for me,” 6 participants (26%) responded negatively (“Nah shit” or “Pfft”), 7 participants (31%) responded positively (“It’s OK” or “Love it!!!”), and the largest proportion of students (10/22, 43%) remained neutral (“Meeh”). While many students did not think the content was personally relevant, most remained interested in participating and providing feedback on the prototype itself.

### Aesthetically Appealing

Participants also gave feedback on the design and branding of The Gist, with the overall look and feel of the application appealing well to young people who took part in the evaluation. In total, 83% of participants (25/30) responded either “It’s ok” or “I love it!!!” to the statement “The Gist branding is hot!” signifying significant approval of the website aesthetic and imagery.

Participants also indicated that they enjoyed the use of bright colours and specifically approved of the retro-90s styling which simultaneously appealed to a wide range of young people and created a nostalgic and safe feeling whilst using the site.

Comic book aesthetic is something that everyone can enjoy

I really enjoy the colours and art style: it makes me feel safe and nostalgic somehow

**Figure 2.**
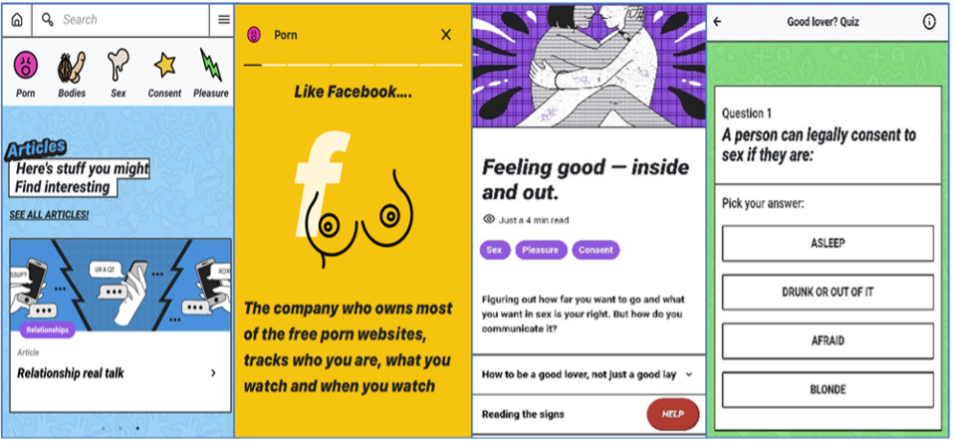
Features of The Gist, a digital prototype for pornography education. Panels show screenshots of the home page, Get Facts, article,and quiz.

For some students, the visual depiction of genitalia, sex, and pornography (Figure 2) was seen as unnecessary and excessive at times, and may have worked to dissuade apprehensive students from engaging with the website.

Some of it is too much, this one that indicates cum in the Get Facts section, it’s not necessary

### Safe and Inclusive

#### User Safety

When asked about how safe they felt using The Gist website, the majority (19/23, 83%) of participants responded either “It’s ok” or “I love it!!!.” Participants particularly enjoyed the ability to browse The Gist anonymously, especially due to the highly sensitive nature of The Gist’s content and associated sexual health topics.

Safe place to get information that you might not want to ask someone about

I like that its anonymous, you don’t need an account to log in

Participants were also asked if they would feel comfortable accessing The Gist website in public spaces such as on a train or tram, with a slight majority (12/22, 54%) responding with either “It’s OK” or “I love it!!!.” Those who felt less safe using The Gist expressed that these sentiments were largely driven by feelings of being unsafe on the internet more generally due to concerns of data monitoring and internet privacy rather than factors specifically related to The Gist website.

### Inclusion of LGBTQIA+ Themes

In general, the content and language used throughout The Gist was seen as inclusive, particularly by those participants who identified as LGBTQIA+. Participants appreciated the nongendered wording used across the site, and the inclusive approach to sex and pornography use amongst gender and sexually diverse people.

It’s not gender specific which is good

Several participants, however, did express a desire to see greater inclusion of LGBTQIA+ themes, and for content to appear more obviously inclusive of sexual and gender diverse young people. This included having a dedicated LGBTQIA+ filter or tab section with relevant content rather than just having inclusive language and content spread throughout the website.

Make it more trans/non-binary (like a language filter). Have LGBTQIA+ filter e.g.: I click trans + all the content is trans-based

fully inclusive – could be more obvious. Have LGBTQIA+ section

### Differences in Cultural Perspectives Toward Sex and Pornography

Discussions with participants who were recent migrants or from culturally and linguistically diverse backgrounds suggested that cultural understandings played a significant role in how they experienced The Gist website and its related content. Some young people indicated that in their culture or religion, acts of intimacy and sex were typically seen as only appropriate within marriage, and therefore influenced their view on the necessity of a sexual health education and pornographic literacy platform.

Several other participants were willing to discuss issues surrounding healthy relationships and consent, but were less comfortable discussing sexual behaviors and pornography. Even though some of these participants felt overwhelmed at times during the first workshop, all participants returned to the second workshop and appeared to be comfortable accessing The Gist and fully participating in the remaining activities. This highlights the importance of giving participants’ time to develop rapport and to “warm up” to discussing sensitive topics during co-design processes.

Feel uncomfortable when quiz answers mention sex in relationships

Confronting images

### Potential of The Gist to Positively Impact Other Young People

Participants were asked whether they believed The Gist had the potential to impact other young people’s knowledge, attitudes, and confidence around healthy relationships, sexual health issues, and pornographic literacy. Most participants perceived that it would be an effective sexual health resource for other young students.

I like it and think it would be helpful and informative for teens and young adults.

…it is a web site that helps young people no matter the gender/ethnicity to understand more about erotic matters.

However, once again participants reflected that the ability of The Gist to impact other young people’s sexual health and pornographic literacy was dependent on its relevance to the user, with greater efforts needed to provide appropriate information to different ages and knowledge levels.

It depends on what kinda age group that you want, I think more detail would be good if you want older people.

I wish that it appealed to an older audience as well as a younger one

When asked if “The Gist will make people want to watch porn,” 63% of participants (14/22) disagreed with the statement, and only 1/22 agreed (“probs”). Participants stated that The Gist was more likely to reduce the stigma associated with watching pornography, and therefore makes young people feel less “scared” about watching pornographic material.

Using the website was easy to read & understand the gist will not make you want to watch porn it will instead give you an insight what porn really is!

I think it might make people feel comfortable not scared to watch porn

## Discussion

### Principal Findings

The Gist prototype was developed through a multistage co-design process, which includes this evaluation as part of the iteration and refinement stage. A key criticism made by participants was that much of the Gist’s content and messaging was too text-heavy, particularly when providing users with in-depth or supplementary information on key sexual health topics. Feedback from participants emphasised this as a barrier to engagement, with many expressing their dislike of the articles feature and other text-based elements of the website. This is despite this feature being intentionally designed based on previous research of young people’s user experiences and online interaction patterns, as well as feedback from the initial prototype co-design stage, in which young people supported the progressive disclosure of supplementary text information [4]. This highlights the importance of a multistage co-design and prototype refinement process, with further work needed to optimize the Gist’s articles for enhanced user engagement and acceptability. In contrast, the interactive learning activities such as the “Debunked” (myth-busting) and “quiz” features were amongst the most used and well-liked on The Gist platform. This affinity for highly interactive, image-based activities is unsurprising, given the well documented preference for kinaesthetic and visual learning styles amongst this cohort [28]. Additional efforts are therefore needed to further adapt key supplementary health information on The Gist website to more appropriate styles, including interactive activities, image-based content, and audio-visual materials. However, consideration must also be given to the resources associated with producing and maintaining such a large volume of supplementary content, including the capacity to update this information as young people’s information needs change.

Despite enthusiastic engagement from young people during the evaluation workshops, website analytical data indicated that few participants accessed The Gist outside of the classroom setting. It was also noted that many participants preferred to explore The Gist collectively with fellow classmates, rather than as an individual learning exercise. This suggests that young people are potentially more likely to engage with The Gist as a sexual health and pornography literacy resource within a facilitated and supported learning environment. Consequently, this poses important questions for future iterations of The Gist, including how to strengthen independent engagement with the website, and whether The Gist as a standalone digital platform is a sufficient intervention to meet the needs of this population group. As noted by the research team in earlier co-design stages [4], previous international research has also shown that while young people appreciate the availability of sexual health materials online, they often seek out the guidance of trusted adults and authority figures to help them make sense of this information, and to determine its validity [29,30]. Thus, consideration should be given to the overall structure of The Gist as an educational intervention, and whether alternative delivery methods, (such as a potential hybrid model involving face-to-face sessions at schools, community sites, or services) are more effective approaches to supporting the sexual development and wellbeing of structurally marginalised young people.

This study adds to limited research into the development and evaluation of pornography education. A recent systematic review identified only 2 education programs that addressed pornography as a major focus [10]. Neither have described in depth the process of designing and developing the program.

### Wider Implications

As so called “digital natives,” there is a widespread perception of young people as highly proficient consumers and navigators of digital information. However, digital literacy is associated with socio-economic status, with online health information less accessible and interpretable to young Australians of lower socioeconomic standing [31,32]. The principal findings of this evaluation, therefore, provide valuable insights into how structurally marginalized young people perceive The Gist as a digital health resource, including key user interface and design preferences, information needs, and online learning requirements.

As pornography literacy programs emerge within mainstream education settings throughout Australia [7], it is important to recognize that young people are not homogenous, and their information needs are likely to differ despite similarities in demographic characteristics. Even amongst our small cohort of structurally marginalized young people, there was a wide variation in experience levels, previous knowledge, and attitudes toward sexual and pornographic themes. These differences reflect the fact that adolescent sexual development is a highly individualised process, shaped by a range of biological, psychological, and social factors [33]. For our participants, this also meant that the perceived relevance and desirability of the Gist prototype differed depending upon their own set of unique personal circumstances. Participants from culturally and linguistically diverse backgrounds also shared how their differing cultural perspectives and understandings of sex and pornography shaped their experiences with the prototype and receptiveness to the key health messages within. Future pornography literacy programs, therefore, need to ensure that the information and content presented is suited to, and tailorable for a wide range of experience and knowledge levels, even within similar demographic cohorts, and is offered in a way that is inclusive of all sexuality, gender, and culturally diverse people.

This evaluation has developed recommendations for further prototype refinements. It is vital that additional testing be carried out following these modifications to ensure The Gist remains highly relevant to structurally marginalized young people seeking accessible sexual health and pornography information. Future research is also needed to assess the impact of The Gist on young people’s knowledge, attitudes, and behaviors, and to assess its effectiveness as a standalone sexual health and pornography literacy intervention.

### Limitations

There are several limitations in which the findings described above should be considered. Study participants were recruited through a single service provider, and were therefore already familiar with other participants and supporting staff members. While this familiarity may have helped some participants to feel more comfortable during workshop sessions, it also introduces the potential for social and group biases. In the presence of their peers and teachers, participants may have sought to conform to the dominant group narrative by offering opinions that felt more socially acceptable. The sensitive nature of the research content may have further exacerbated this, with participants potentially hesitant to share their own personal experiences with sex and pornography. Similarly, while facilitators emphasized the need for critical and honest feedback, participants may have felt inclined to provide positive assessments of the website as an unintended courtesy to the research team, particularly as 2 of the facilitators had been involved in development of the prototype. The design of the evaluation and the analysis of data was conducted by 3 researchers who were not directly involved in development of the prototype; however, they were close colleagues of those who did develop the prototype.

Young people were also free to engage with The Gist prototype as much or as little as they liked, however, not all participants attended both workshops, meaning that exposure varied. During the workshops, facilitators provided additional prompts and guidance only as needed, and there was no prerequisite that all elements of The Gist be explored and evaluated by participants. While this flexibility is an integral part of co-design research with structurally marginalized cohorts, it also means that again, participants may have had variable experiences with The Gist website, leading to differing assessments of the prototype. Similarly, as The Gist was an early prototype, access was only available through a password protected URL link, further limiting potential student engagement with the site. Finally, the evaluation did not use any validated instruments or apply techniques to enhance the validity or credibility of data analysis.

### Conclusion

Amid growing concerns over the potential impact of pornography on the sexual development of young people, digital resources such as The Gist are emerging as potential alternatives to contemporary interventions seeking to combat these concerns. Research is still needed to determine the efficacy and impact of digital pornography literacy resources on young people’s sexual attitudes, beliefs, and behaviors. The findings from this formative evaluation highlight the potential for creating engaging and highly pertinent online platforms for pornographic literacy, specifically tailored to the distinctive requirements of structurally marginalized young people. However, low engagement with the prototype outside of facilitated settings suggests that a hybrid delivery model that incorporates face-to-face elements may be preferable. This study emphasizes that intervention development is a long-term process requiring multiple iterations of prototype evaluation and refinement.

## Supplementary material

10.2196/65859 Multimedia Appendix 1 Workshop aims.

10.2196/65859 Multimedia Appendix 2 Workshop activities.

10.2196/65859 Multimedia Appendix 3 Workshop survey results.

## Abbreviations

LGBTQIA+: lesbian, gay, bisexual, transgender, queer, questioning, intersex, asexual
STI: sexually transmitted infection
VicHealth: Victorian Health Promotion Foundation
