# Supplementary material for: A Digital Pornography Education Prototype Co-Designed With Young People: Formative Evaluation
Source: JMIR Form Res. 2025 Mar 4;9:e65859. doi: 10.2196/65859 (PMC11896553; doi:10.2196/65859)
Supplement: Multimedia Appendix 1 [file formative-v9-e65859-s001.docx]

# Appendix 2: Workshop Activities

| **Workshop Activity** | **Activity Description** | **Activity Used** |
| --- | --- | --- |
| Introduction | Brief introduction to the workshop aims | First workshop |
| Icebreaker | Brief introductory activity designed to acquaint students with workshop facilitators and content. Students were asked to create a ‘superhero’ pseudonym name that can be used throughout the workshops to minimise feelings of personal awkwardness and facilitate better engagement. | First workshop |
| Explore the Gist | Young people were provided with their first interaction with The Gist website, and were given free time to explore the resources different features for a few minutes. Students were then presented with different tasks to guide students through navigating the website. Students were asked to find and interact with different features such as quizzes, quick facts, and articles. This ‘WebQuest’ style activity was designed to get students familiar with the website and its key features. | First workshop |
| First Impressions | After students were acquainted with The Gist website, a worksheet was distributed that asked a series of questions designed to understand the desirability and relevance of The Gist for structurally marginalised young people. This activity sought to gauge student’s initial impressions of the website, including how it looked, how it made them feel, and any other first impressions. | First workshop |
| Have your say | After having viewed The Gist website, students were presented with a series of statements on a large piece of paper pinned to the wall. Each student placed a sticker on a response scale from 1 – 5 rating their overall agreement to the statement. Students were also encouraged to write a short statement on their sticker explaining their rating. | Both workshops |
| Control Arc Scenario | Students were presented with a frame-by-frame style worksheet with characters present in the first and last scenes, and asked to fill in the missing frames using their own stick-figure drawings or speech bubbles, illustrating how the characters interacted between the supplied scenes. The various scenarios were designed to elicit responses to notions of consent, and how to seek and/or give consent in different circumstances. Two different versions of the same scene were completed, with students asked to depict how they believe the scenario would occur in real life compared to how the same scenario would occur in pornographic content. Facilitators then lead a discussion highlighting the difference and similarities between the two different versions, and explored how this would impact young people’s perception of consent in real life.    In the second workshop, students were asked to make explicit reference to The Gist, and illustrate how the ‘real life’ characters would change their behaviour because of their access to The Gist prototype.    The Control Arc Scenario was designed to explore the influence of The Gist on participants’ knowledge, attitudes, and confidence around respectful and healthy relationships as well as their sexual health and wellbeing literacy. | Both workshops |
| Quizzes | Two online quiz activities were developed to better gauge student’s overall knowledge of the key sexual health themes explored by The Gist. The first quiz ‘What I Think About Sex’ explored student’s perceptions and understandings of sex, masturbation, and other sexual activities, whilst the second quiz ‘I Feel Confident’ sought to gauge student’s attitudes and understandings towards sex, pornography, relationships and consent. Students were instructed to find a quite spot nearby to complete the quizzes anonymously on their personal devices before rejoining the group. | Both workshops |
| Card sort | A series of behaviours relating to sex, consent and relationships were printed onto cards, with students tasked with sorting the set into two piles, one for behaviours they considered healthy and another for behaviours they considered unhealthy. Facilitators then worked their way through each pile, asking students for the reasonings behind their assessments of the behaviour and relating it back to scenarios young people may relate to. Once again, this activity sought to measure student’s knowledge, attitude and confidence around sexual health behaviour and actions. | Both workshops |
| Gist Refresher | The Gist Refresher activity was used at the beginning of the second workshop to reacquaint participants with The Gist prototype. Students were asked to find and note down on a worksheet particular features of The Gist website that   1. They liked the most 2. Annoyed them the most 3. They wouldn’t use   This activity aimed to assess the useability of The Gist following previous engagement with the prototype, and to identify any missing features deemed particularly useful and/or desirable.    Students were also asked to find an article on The Gist website that they enjoyed, re-read it to themselves before noting down a key fact they learned and shared it with the class. This task also assessed the usability of the prototype and student’s acceptability and understanding of key messages and content delivered. | Second workshop |
| Wishlist | This activity sought direct feedback from student’s regarding the desirability and relevance of The Gist as a prototype resource for structurally marginalised young people. Students were asked to brainstorm and write down on butchers’ paper, any features, content, features or other elements of The Gist that they wish they could change or add to The Gist. | Second workshop |
| Marketing Plan | Students were asked to prepare a ‘marketing plan’ for The Gist with the premise of scaling-up and marketing the prototype resource to the broader Australian youth population. Students were asked to define a target audience, key messages that they would convey, why their target audience should use The Gist as an educational resource, and any strategies they would adopt to promote the use of the website. Students were also asked to choose and customise a template of Instagram ads, YouTube videos and launch events before presenting their ideas back to the group. This activity sought to gain insights from participants on the most desirable and receptive means of promoting The Gist amongst young people like themselves. | Second workshop |
