# Supplementary material for: A Digital Pornography Education Prototype Co-Designed With Young People: Formative Evaluation
Source: JMIR Form Res. 2025 Mar 4;9:e65859. doi: 10.2196/65859 (PMC11896553; doi:10.2196/65859)
Supplement: Multimedia Appendix 3 [file formative-v9-e65859-s003.docx]

# Appendix 3: Workshop Survey Results

| **Question/Statement and (attribute measured)** | **Response** | **n** | **%** |
| --- | --- | --- | --- |
| “The Gist is easy to use”  (usability and accessibility) | Love it!!! | 12 | 60% |
|  | It’s OK | 5 | 25% |
|  | Meeh | 3 | 15% |
|  | Pfft | 0 | 0% |
|  | Nah shit | 0 | 0% |
|  | Total | 20 | 100% |
|  |  |  |  |
| “I like the way articles break down the information”  (usability and accessibility) | Love it!!! | 11 | 52% |
|  | It’s OK | 6 | 29% |
|  | Meeh | 3 | 14% |
|  | Pfft | 1 | 5% |
|  | Nah shit | 0 | 0% |
|  | Total | 21 | 100% |
|  |  |  |  |
| “The articles are easy to read”  (usability and accessibility) | Love it!!! | 11 | 52% |
|  | It’s OK | 9 | 43% |
|  | Meeh | 1 | 5% |
|  | Pfft | 0 | 0% |
|  | Nah shit | 0 | 0% |
|  | Total | 21 | 100% |
|  |  |  |  |
| “The Gist is definitely something I would use”  (desirability and relevance) | Love it!!! | 4 | 18% |
|  | It’s OK | 11 | 50% |
|  | Meeh | 3 | 14% |
|  | Pfft | 3 | 14% |
|  | Nah shit | 1 | 5% |
|  | Total | 22 | 100% |
|  |  |  |  |
| I learnt some cool stuff from The Gist  (desirability and relevance) | Love it!!! | 11 | 50% |
|  | It’s OK | 5 | 23% |
|  | Meeh | 5 | 23% |
|  | Pfft | 1 | 5% |
|  | Nah shit | 0 | 0% |
|  | Total | 22 | 100% |
|  |  |  |  |
| “The Gist feels like it was made for me”  (desirability and relevance) | Love it!!! | 2 | 9% |
|  | It’s OK | 5 | 22% |
|  | Meeh | 10 | 43% |
|  | Pfft | 3 | 13% |
|  | Nah shit | 3 | 13% |
|  | Total | 23 | 100% |
|  |  |  |  |
| “The Gist branding is hot!”  (desirability and relevance) | Love it!!! | 19 | 63% |
|  | It’s OK | 6 | 20% |
|  | Meeh | 2 | 7% |
|  | Pfft | 2 | 7% |
|  | Nah shit | 1 | 3% |
|  | Total | 30 | 100% |
|  |  |  |  |
| “I feel safe on The Gist”  (safety and inclusivity) | Love it!!! | 6 | 26% |
|  | It’s OK | 13 | 57% |
|  | Meeh | 3 | 13% |
|  | Pfft | 1 | 4% |
|  | Nah shit | 0 | 0% |
|  | Total | 23 | 100% |
|  |  |  |  |
| “I would totally play Debunked on the train/tram”  (desirability and relevance) | Love it!!! | 6 | 27% |
|  | It’s OK | 6 | 27% |
|  | Meeh | 4 | 18% |
|  | Pfft | 2 | 9% |
|  | Nah shit | 4 | 18% |
|  | Total | 22 | 100% |
|  |  |  |  |
| “The Gist will make people want to watch porn”  (potential to impact) | Defs | 0 | 0% |
|  | Probs | 1 | 5% |
|  | Meeh | 7 | 32% |
|  | Not really | 5 | 23% |
|  | Defs not | 9 | 41% |
|  | Total | 22 | 100% |
